# A comparison of the effects of monotherapy with rosuvastatin, atorvastatin or ezetimibe versus combination treatment with rosuvastatin-ezetimibe and atorvastatin-ezetimibe on the integrity of vascular endothelial cells damaged by oxidized cholesterol

**DOI:** 10.1371/journal.pone.0256996

**Published:** 2021-09-07

**Authors:** Mateusz Niedzielski, Marlena Broncel, Paulina Gorzelak-Pabiś, Ewelina Woźniak

**Affiliations:** Department of Internal Diseases and Clinical Pharmacology, Laboratory of Tissue Immunopharmacology, Medical University of Lodz, Lodz, Poland; Xiangtan University, CHINA

## Abstract

Dyslipidemia, atherosclerosis, and cardiovascular events can be prevented, or treated, using statins, alone or in combination with ezetimibe. The aim of the study was to compare the direct pleiotropic effects of two commonly-used statins (atorvastatin, rosuvastatin), ezetimibe and their combinations on endothelial cells damaged by oxidized cholesterol. HUVEC cultures were stimulated for 20 hours with atorvastatin (5 μM; 2793 ng/mL), rosuvastatin (10 μM; 4815 ng/mL), ezetimibe (1.22 μM; 500 ng/mL), atorvastatin plus ezetimibe (5 μM + 1.22 μM; 2793 ng/mL + 500 ng/mL) and rosuvastatin plus ezetimibe (10 μM + 1.22 μM; 4815 ng/mL + 500ng/mL) in separate groups, with or without 25-hydroxycholesterol pre-incubation (24.83 μM; 10 μg/mL; four hours then washout). HUVEC integrity was measured in the RTCA-DP xCELLigence system. The mRNA expression and protein levels of ZO-1, OCLN, ICAM-1 were analyzed by real-time PCR and ELISA. Pre-incubation with 25-OHC resulted in decreased endothelial cell integrity (p<0.001), decreased expression of ZO-1 mRNA (p<0.05) and protein levels (p<0.05), OCLN mRNA (p<0.05) and protein levels (p<0.05) and increased ICAM-1 mRNA (p<0.001) and protein levels (p<0.001) compared to the control group. Incubation with rosuvastatin (12h p<0.01; 24h p<0.001) and atorvastatin (only 12h p<0.05) restored HUVEC integrity. Subsequent incubation with rosuvastatin increased ZO-1 mRNA (p<0.001) and protein (p<0.001) levels. Subsequent addition of ezetimibe increased ZO-1 mRNA level (p<0.001) but not protein level. Furthermore, only incubation with rosuvastatin increased OCLN mRNA (p<0.05) and protein (p<0.05) levels. In each drug-stimulated group, both ICAM-1 mRNA and protein levels were reduced after initial incubation with oxysterol (p<0.05). 25-hydroxycholesterol disrupts endothelial integrity, decreases the mRNA and protein levels of tight junction, and increases those of intercellular adhesion molecules. Both rosuvastatin and atorvastatin can improve endothelial integrity, but only rosuvastatin can completely abolish the effect of oxysterol. The combination of statins with ezetimibe has less direct effect on the endothelial barrier than the statins alone.

## Introduction

Cardiovascular disease (CVD) is the most common cause of deaths in high-income countries. One of the most important risk factors for CVD is atherosclerosis, an inflammatory disease of the vascular wall which leads to atherosclerotic plaque formation and vascular obstruction. The onset of atherosclerosis is provoked by a high concentration of oxidized low-density lipoproteins (oxLDLs), which causes a complex inflammatory process, including increased endothelial permeability, monocyte migration, macrophage activation and vascular smooth muscle cell (VSMC) migration into the intima [1].

The atherosclerosis process is influenced by 25-hydroxycholesterol (25-OHC), an endogenous oxidative cholesterol derivative [2]. Serum 25-OHC level increases after a high-fat meal and is significantly higher in hypercholesterolemic than normocholesterolemic serum [3–5]. At the level of the vascular wall, 25-OHC exhibits pro-inflammatory properties, which include endothelial dysfunction, increased oxidative stress and pro-inflammatory cytokines [6]. Moreover, Gold *et*. *al* [7] (2012) showed that the inhibition of 25-OHC synthesis attenuates the formation of macrophage foam cells and inhibits atherosclerosis. These findings confirm that 25-OHC is a key element in initiating atherosclerosis and show how important it is to properly understand the effects of 25-OHC on the vascular wall.

Statins, 3-hydroxy-3-methyl-glutarylCoA reductase inhibitors, are widely used in clinical practice to reduce serum LDL, treat atherosclerosis and prevent CVD. According to the 2019 guidelines of the European Society of Cardiology (ESC) and the European Atherosclerosis Society (EAS) [8], low-density lipoprotein (LDL) cholesterol levels should be lowered as much as possible to prevent cardiovascular disease. It is recommended that very high-risk patients should achieve both a goal LDL-C level of <55 mg/dL and at least 50% reduction from baseline LDL-C levels. In high-risk patients, the LDL-C goal is <70 mg/dL or and at least 50% reduction from baseline LDL-C levels. The guidelines in high and very high-risk patients recommend high-intensity statin—atorvastatin (40–80 mg) or rosuvastatin (20–40 mg) or combination therapy ezetimibe with statins in cases of statin intolerance or insufficiency [9]. Beside the hypolipidemic effect, statins also have a pleiotropic anti-inflammatory effect, which can result in additional benefits to patients in high-risk groups [10]. Despite many studies, we still do not know whether combined treatment with statins and ezetimibe offers greater pleiotropic properties than statin monotherapy [10].

The present study examines the effect of statin, used alone or in combination with ezetimibe, on restoring the integrity of human umbilical vein endothelial cells (HUVEC) after 25-OHC incubation, by assessing gene expression and protein levels of tight junction proteins (*ZO-1*, *OCLN*) and intercellular adhesion molecule (*ICAM-1*).

## Results

### Analysis of integrity of the human endothelial cells (HUVEC) monolayer by RTCA-DP

RTCA-DP was used to monitor dynamic changes in the barrier properties of endothelial cell (EC) monolayers after reaching 80–90% confluence (24 hours after seeding the cells). Data for cell adherence were normalized (nCl) before incubation with compounds.

**Fig 1:**
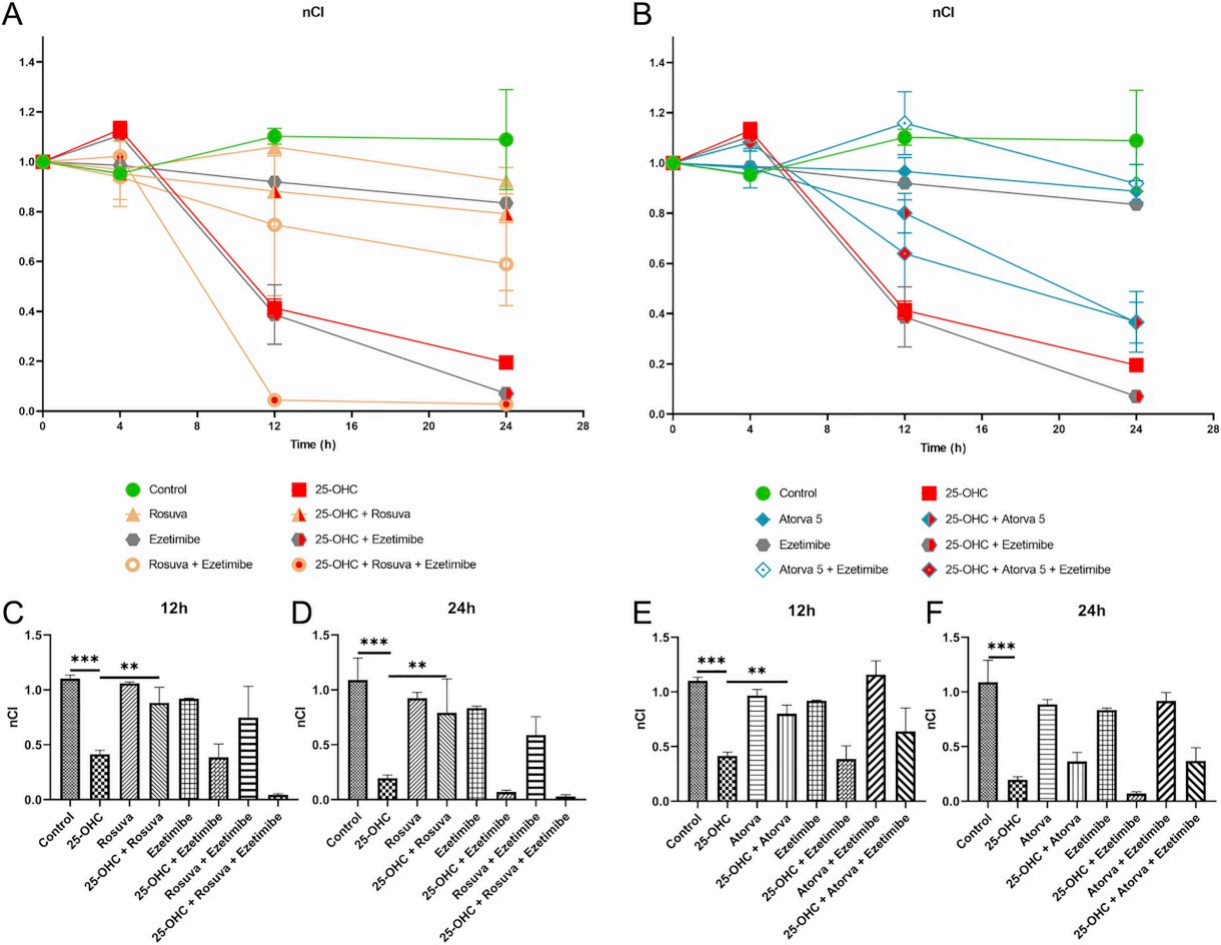
Changes in the cell index of HUVEC damaged by 25-OHC (24.83 μM; 10 μg/mL) and then stimulated with rosuvastatin (10 μM; 4815 ng/mL), atorvastatin (5 μM; 2793 ng/mL), ezetimibe (1.22 μM; 500 ng/mL), ezetimibe in combination with rosuvastatin and ezetimibe in combination with atorvastatin. In the (A-B) graph, Time 0 shows the normalized cell index of HUVEC after 24 hours of incubation with the medium. The cells were then stimulated with 25-OHC for four hours. Following this, oxidized cholesterol was removed and the cells were stimulated with the selected drugs. The cell index results at 12 hours (C, E) and 24 hours (D, F) are shown separately for rosuvastatin/ezetimibe (C-D) and atorvastatin/ezetimibe (E-F). Each experimental group included a control group without incubation (control) or without pre-incubation of 25-OHC, i.e. drug incubation only. The changes in cell index were measured in real time using the RTCA-DP xCELLigence system. The number of cells scored for each well in a 16-well plate was 10 000. Mean ± SD calculated from three biological replicates. Significant differences from negative controls are indicated by *P < 0.05; **P < 0.01; ***P < 0.001. Statistical analysis was conducted using one-way ANOVA and *post hoc* Tukey’s test.

At a concentration of 10 μg/mL (24.83 μM), 25-hydroxycholesterol (after four-hour incubation and washout) administration decreased the integrity of the HUVEC monolayer after 12 hours (0.41 ± 0.04, p<0.001) and 24 hours (0.20 ± 0.03, p<0.001) (Fig 1A–1F). Incubation with 25-OHC (10 μg/mL; four hours then washout) followed by incubation with atorvastatin (5 μM; 2793 ng/mL) resulted in an increase in the cell index, but only in the 12-hour experiment (0.80 ± 0.08, p <0.05) (Fig 1B and 1E). Finally, only rosuvastatin (10 μM; 4815 ng/mL) was able to restore the cell index to a baseline level after initial incubation with 25-OHC (10 μg/mL; 4 hours then washout) at both 12 hours (0.88 ± 0.14, p <0.01, Fig 1A and 1C) and 24 hours (0.79 ± 0.31, p <0.01, Fig 1A and 1D).

Incubation of endothelial cells with ezetimibe alone, and in combination with statins, did not result in greater cell integrity after 25-OHC preincubation (10 μg/mL; four hours then washout), neither after 12 hours (Fig 1C and 1E), nor 24 hours (Fig 1D and 1F).

### Analysis of gene expression and protein levels

Gene expression was measured with real-time reverse transcription polymerase chain reaction (RT-PCR). The protein levels were measured with ELISA. mRNA and supernatants were isolated from HUVEC after 25-hydroxycholesterol (25-OHC) pre-incubation and drug incubation, at 24 hours of the experiment. Pre-incubation with 25-OHC (24.83 μM; 10 μg/mL) lasted four hours and was performed after 24-hour incubation of HUVEC in medium. Following this, the oxysterol was washed out and cells were stimulated for a further 20 hours with medium (control group), atorvastatin (5 μM; 2793 ng/mL), rosuvastatin (10 μM; 4815 ng/mL), ezetimibe (1.22 μM; 500 ng/mL) and combinations of atorvastatin with ezetimibe, rosuvastatin with ezetimibe. mRNA and supernatants were isolated after 20 hours of drug incubation, which corresponds to 24 hours of the experiment (four hours of pre-incubation with 25-OHC + 20 hours of drug incubation). The times of incubation with individual substances were selected based on the observation of the integrity of the HUVEC in the RTCA-DP system.

### The expression of the gene and protein levels of tight junction proteins (*ZO-1*, *OCLN*) and intercellular adhesion molecules (*ICAM-1*)

**Fig 2:**
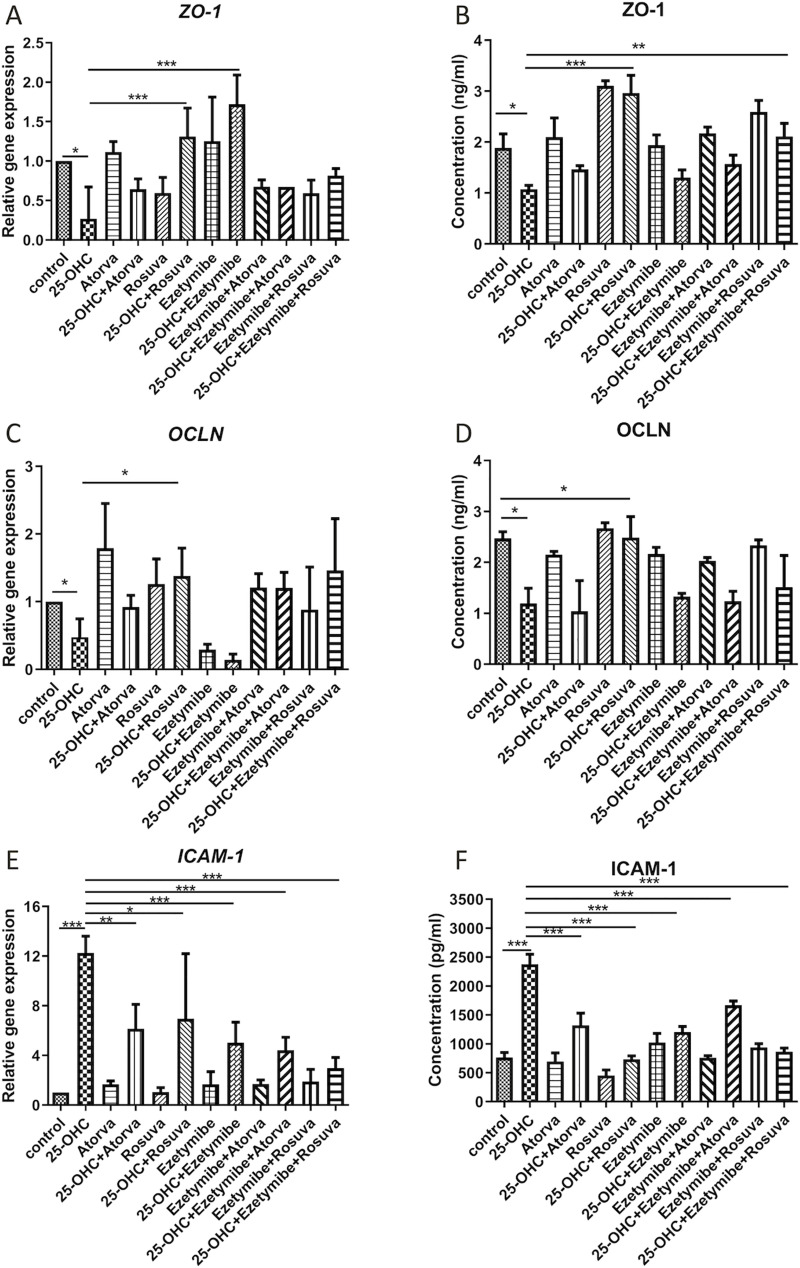
Relative gene expression and protein levels of (A-D) tight junction and (E, F) intercellular adhesion proteins in HUVEC prestimulated with 25-OHC (24.83 μM; 10 μg/mL; four-hour incubation followed by removal of oxysterol) and stimulated for 20 hours with rosuvastatin (10 μM; 4815 ng/mL), atorvastatin (5 μM; 2793 ng/mL) and ezetimibe (1.22 μM; 500 ng/mL), as well as ezetimibe in combination with rosuvastatin, and ezetimibe in combination with atorvastatin. Each experimental group included a control group without pre-incubation of 25-OHC. The level of mRNA was measured by real time Polymerase Chain Reaction (RT-PCR). The protein levels were measured by ELISA. The number of cells scored for each well was 40 000. Mean ± SD calculated from four biological replicates. Significant differences from negative controls are indicated by *P < 0.05; **P < 0.01; ***P < 0.001. Statistical analysis was conducted using one-way ANOVA and *post hoc* Tukey’s test.

Four-hour incubation with 25-hydroxycholesterol at a concentration of 10 μg/mL (24.83 μM) resulted in decreased levels of Zonula Occludens-1 mRNA (*ZO-1* gene) (0.26 ± 0.40, p<0.05) and protein (1.08 ± 0.07, p<0.05). Following this, ZO-1 mRNA levels were restored by the subsequent addition of rosuvastatin (10 μM; 4815 ng/mL; 1.31 ± 0.36, p<0.001) and ezetimibe (1.22 μM; 500 ng/mL; 1.72 ± 0, 37, p<0.001). The protein levels were restored by subsequent addition rosuvastatin (10 μM; 4815 ng/mL; 2.96 ± 0.35, p<0.001) and combination of rosuvastatin with ezetimibe (10 μM + 1.22 μM; 4815 ng/mL + 500 ng/mL; 2.11 ± 0.26, p<0.01). This effect was not observed with atorvastatin (5 μM; 2793 ng/mL) incubation, nor with the combinations of atorvastatin and ezetimibe (5 μM + 1.22 μM; 2793 ng/mL + 500 ng/mL) (Fig 2A and 2B).

The incubation with 25-hydroxycholesterol (10 μg/mL; four hours) also decreased the concentration of occludin mRNA (*OCLN* gene) (0.34 ± 0.08, p<0.05) and protein (1.19 ± 0.30, p<0.05). Only subsequent rosuvastatin (10 μM; 4815 ng/mL; next 20 hours) incubation was able to significantly increase gene expression (1.38 ± 0.41, p<0.05) and protein levels (2.49 ± 0.41, p<0.05). No such effect was observed following incubation with atorvastatin (5 μM; 2793 ng/mL), ezetimibe (1.22 μM; 500 ng/mL) or combinations of atorvastatin and ezetimibe (5 μM + 1.22 μM; 2793 ng/mL + 500 ng/mL) or rosuvastatin and ezetimibe (10 μM + 1.22 μM; 4815 ng/mL + 500 ng/mL) (Fig 2B and 2C).

The administration of 25-hydroxycholesterol (10 μg/mL; four hours) greatly increased the concentration of *ICAM-1* gene mRNA (12.25 ± 1.35, p<0.001) and protein (2375.0 ± 173.2, p<0.001). Expression decreased after the subsequent addition of atorvastatin (5 μM; 2793 ng/mL; mRNA: 6.16 ± 1.96, p<0.01; protein: 1321.0 ± 209.3, p<0.001), rosuvastatin (10 μM; 4815 ng/mL; mRNA: 6.95 ± 5.24, p<0.05; protein: 733.3 ± 57.7, p<0.001), ezetimibe (1.22 μM; 500 ng/mL; mRNA: 5.02 ± 1.65, p<0.001; protein: 1204.0 ± 93.8, p<0.001) and the following combinations into the cellular environment: atorvastatin with ezetimibe (5 μM + 1.22 μM; 2793 ng/mL + 500 ng/mL; mRNA: 4.41 ± 1.05, p<0.001; protein: 1667.0 ± 72.2, p<0.001) and rosuvastatin with ezetimibe (10 μM + 1.22 μM; 4815 ng/mL + 500 ng/mL; mRNA: 2.98 ± 0.85, p<0.001; protein: 862.5 ± 65.0, p<0.001) (Fig 2D–2F).

## Discussion

This study is the first to investigate the effects of oxidized cholesterol (25-hydroxycholesterol), statins (rosuvastatin, atorvastatin) and ezetimibe, both alone and in combination (i.e. atorvastatin with ezetimibe, rosuvastatin with ezetimibe) on endothelial cells.

It was found that neither 25-hydroxycholesterol, rosuvastatin, atorvastatin, ezetimibe nor the tested combinations of ezetimibe with statins (at the concentrations used in this experiment) affected HUVEC viability. These results are confirmed by literature data. Gorzelak-Pabis *et*. *al*. (2019) [11] obtained a similar result after incubation of endothelial cells with a mixture of palmitic acid (800 μM = 205.1 μg/mL) and 25-hydroxycholesterol (24.83 μM; 10 μg/mL). Zhao *et al*. (2019) [12] report that atorvastatin had a dose-dependent effect on HUVEC apoptosis. Although no such effect was observed at a concentration of 0.7 μM (391 ng/mL), it was found that cell viability was decreased and necrosis increased at 7, 35 and 70 μM (3910, 19 551 and 39 102 ng/mL). Literature data not only confirms that rosuvastatin does not induce apoptosis, but also it has a protective effect on HUVEC stimulated with ox-LDL (200 μg/mL) at concentrations of 0.01, 0.1 and 1 μM (4.8, 4.82 and 481.5 ng/mL) [13]. Similarly, Qin *et al*. (2018) [14] indicate that ezetimibe at concentrations of 1, 2.5, and 5 μM (409.4, 1023.5 and 2047 ng/mL) can prevent the decrease in HUVEC viability caused by ox-LDL (100 mg/dL).

25-hydroxycholesterol is an oxidative derivative of cholesterol involved in several processes, including inflammation and vascular proliferation, which promote development of atherosclerosis. Our current findings show that 25-hydroxycholesterol (24.83 μM; 10 μg/mL) can effectively lower the HUVEC cell index observed in RTCA-DP, demonstrating reduced cell integrity. A similar conclusion was presented by Chalubinski *et al*. (2013) [15] in studies on human primary aortic endothelial cells (HAEC) and Caco-2 intestinal epithelial cells, also using the same RTCA-DP monitoring system. They report that 25-OHC (24.83 μM; 10 μg/mL) significantly decreased normalized Cell Index (nCI) of HAEC (after 48 hours of incubation) and Caco-2 (after 10 and 24 hours of incubation).

Our major finding showed that rosuvastatin and atorvastatin increased endothelial integrity damaged by 25-OHC in RTCA-DP. Rosuvastatin, atorvastatin, ezetimibe and combinations of ezetimibe with statins (at the concentrations used in this experiment) did not appear to disturb HUVEC integrity. Moreover, both atorvastatin (5 μM; 2793 ng/mL) and rosuvastatin (10 μM; 4815 ng/mL) contributed to the restoration of HUVEC integrity after 12 hours, following disruption by pre-incubation with oxysterol. However, after rosuvastatin treatment, this effect was also found to persist after 24 hours of incubation. These results are confirmed by literature data. Haidari *et al*. (2012) [16] report that HUVECs incubated with atorvastatin (5 μM; 2793 ng/mL; 16h) and stimulated with IL-1β (10 ng/mL; 30 min) do not form an inter-endothelial gap and present lower migration of human acute monocytic leukemia (THP-1) cells than those stimulated only with IL-1β. In turn, Kathuria *et al*. (2013) [17] showed that rosuvastatin (10 mg/kg/day, p.o.; four-week therapy) significantly improved the integrity of the endothelial cell layers and the endothelial blood vessels of the rat thoracic aorta.

Our study revealed that ezetimibe, either alone or in combination with statins, has no direct effect on the integrity of HUVEC observed in the RTCA-DP system, either with or without initial 25-OHC incubation. This is the first study to analyze the effects of ezetimibe on endothelial cells, so further studies are required. However, the lack of effect observed for ezetimibe may be the reason why, despite many years of research, its pleiotropic effect remains unconfirmed [10].

According to current knowledge [18,19] the inflammatory process may be initiated by an increase in the serum level of oxLDL. Inflammation modulates the expression of two sets of genes: those involved in the intercellular junction and those involved in the inflammatory process. The first group is responsible for the integrity of the endothelium, the second is mainly responsible for the migration of immune cells, macrophage activation and oxidative stress. Both tight junctions and adhesion molecules are involved in maintaining proper endothelial integrity. Therefore, the present study focusses on changes in the expression of genes known to be responsible for tight junctions (*ZO-1*, *OCLN*) and intercellular adhesion molecules (*ICAM-1*).

Our data indicates that incubation with 25-hydroxycholesterol (24.83 μM; 10 μg/mL; four hours) significantly decreases both the expression of the ZO-1 and OCLN genes and the levels of the encoded proteins. The reduction in both tight junction proteins may explain why 25-OHC was able to effectively reduce endothelial integrity. ZO-1 mediates interactions between tight junction proteins such as claudins, adhesive molecules (JAMs), and the adherent junction proteins afadin and α-catenin, while occludin provides stability and performs a barrier function to tight junctions. Labus *et al*. (2014) [20] reports that transfected human brain microvascular endothelial cells (THBMEC) stimulated with IL-1β (10 ng/mL; 72 hours) exhibit increased permeability and lower levels of ZO-1 but no change in OCLN level. Furthermore, Zhang *et al*. (2020) [21] report that palmitate decrease ZO-1 and VE-cadherin protein levels in HUVEC.

Another important finding in our study reveals that rosuvastatin (10 μM; 4815 ng/mL; 20 hours) has a positive effect on tight junctions by increasing the expression of the *ZO-1* and *OCLN* genes previously lowered by pre-incubation with 25-OHC (24.83 μM; 10 μg/mL; four hours followed by washout). Moreover, it is the only drug that was able to increase both zonula occludens-1 and occludin (mRNA and protein), which may explain why rosuvastatin showed the best beneficial effect on HUVEC integrity in the RTCA-DP monitoring system. These results are consistent with literature data. A study of the blood-brain barrier (BBB) in Balb/c mice undergoing middle cerebral artery occlusion surgery followed by reperfusion with rt-PA thrombolytic therapy, with or without prior rosuvastatin (1 mg/kg or 5 mg/kg) administration, found that rosuvastatin treatment was associated with decreased BBB permeability and increased amounts of the occludin, claudin-5 and VE-cadherin proteins [22]. A similar study by Zheng *et al*. found stroke-prone renovascular hypertensive rats (RHRSPs) treated with rosuvastatin (10 mg/kg/day; 12 weeks) had higher levels of zonula occludens-1, claudin-5 and occludin proteins in the corpus callosum compared to untreated rats [23].

Our data shows that neither atorvastatin (5 μM; 2793 ng/mL; 20 hours) alone nor after initial incubation with oxysterol has any effect on the level of ZO-1 or OCLN mRNA. Similarly, Yi *et al*. (2015) [24] confirm that atorvastatin alone (1 μM; 558.6 ng/mL; 12h) has no effect either on the permeability of human arterial endothelial cells (HAEC) or on the expression of the ZO-1 protein.

Our study indicates that ezetimibe (1.22 μM; 500 ng/mL) can increase ZO-1 mRNA levels after pre-incubation with 25-OHC (24.83 μM; 10 μg/mL). However, it cannot restore OCLN gene expression following oxysterol treatment. Ultimately, ezetimibe neither improved nor damaged endothelial integrity. This is the first analysis to show the effect of ezetimibe on the intercellular junctions of the vascular endothelium, and thus confirms previous studies suggesting that it has no or negligible pleiotropic effect. Earlier studies showing the benefits of using ezetimibe alone emphasize the primary effect of the drug on the intestinal absorption of cholesterol. For example, Nochioka *et al*. (2012) [25] found of ezetimibe (10 mg/day) to have a beneficial effect on endothelial function in healthy volunteers, but they underline that this effect was mediated by the inhibition of intestinal absorption of atherogenic cholesterol.

Garcia *et al*. (2016) [26] compared the effect of a high dose of simvastatin (80 mg/day) with a low dose of simvastatin (10 mg/day) in combination with ezetimibe (10 mg/day) on endothelial function in patients. Endothelial function was assessed by a flow-mediated vasodilator (FMV) method measured before and after eight weeks of treatment. The study found that FMV improved similarly in both groups. However, FMV was correlated with the reduction of LDL-C and not with the type of therapy, suggesting no pleiotropic effect. Moreover, a study based on a group of 260 patients with coronary artery disease found that the combination of ezetimibe with a statin results in a greater reduction in the number of both target vessel dysfunction and coronary endothelial dysfunction compared to statin monotherapy [27]. However, these benefits were also associated with a reduction of LDL-C, as the combination therapy group achieved lower serum LDL-C levels. The above study also shows the advantage of lipid-lowering effects over possible pleiotropic effects.

Our findings indicate that atorvastatin in combination with ezetimibe does not affect the mRNA and protein levels of ZO-1 and OCLN proteins. Moreover, taking into account the lack of ezetimibe effect and the strong effect of rosuvastatin, it should be assumed that rosuvastatin is primarily responsible for the increase in the ZO-1 protein level in the “rosuvastatin with ezetimibe” stimulating group. This result suggests that the combination of these drugs has less effect on intercellular junctions than statins or ezetimibe following a single incubation. This finding may confirm that the benefits of combination therapy depend solely on the lipid lowering effect and not on the pleiotropic mechanism.

Intercellular adhesion molecule 1 (ICAM-1) is involved directly in the adhesion of leukocytes to the endothelium and initiates migration through this layer. Elevated ICAM-1 levels intensify this process.

Our findings demonstrate that 25-hydroxycholesterol (10 μg/mL; 4 hours) increases ICAM-1 mRNA levels in HUVEC, which is consistent with literature data. Similarly, Gorzelak-Pabiś *et al*. (2019) [11] found that stimulating HUVECs with a mixture of palmitic acid (800 μM) and 25-OHC (24.83 μM; 10 μg/mL) was associated with significant increase of ICAM-1 mRNA level. Furthermore, Niwa *et al*. (1996) [28] showed that HUVEC stimulated with lipopolysaccharide (LPS; 10 μg/mL) had an increased concentration of ICAM-1 protein compared to the unstimulated group.

Our study also indicates that atorvastatin (5 μM; 2793 ng/mL; 20 hours) lowers the level of ICAM-1 mRNA and protein level in HUVEC prestimulated with 25-OHC (24.83 μM; 10 μg/mL; 4 hours). This is in line with Zhang *et al*. (2018) [29], who report presenting that administration of atorvastatin (5 mg/kg/day) was associated with reduction of all ICAM-1, IL-1β and TNFα in the serum of rabbits, suggesting it has strong anti-inflammatory potential.

Our results show that rosuvastatin can also reduce the ICAM-1 mRNA and protein levels elevated by 25-OHC treatment (24.83 μM; 10 μg/mL; 4 hours) in HUVEC. This finding is consistent with Luo *et al*. (2020) [30]. High doses of rosuvastatin (20 mg/day; eight weeks) can significantly decrease serum levels of ICAM-1, IL-6 and TNFα in patients with ST-segment elevation myocardial infarction (STEMI).

According to our study, ezetimibe (1.22 μM; 500 ng/mL) may decrease the level of ICAM-1 mRNA increased by 25-OHC (24.83 μM; 10 μg/mL; 4 hours) in HUVEC. This result is in line with literature data. Li *et al*. (2018) [31] report that obese rats treated with ezetimibe (10 mg/kg/day; eight weeks) present lower ICAM-1 levels in myocardial tissue than untreated obese rats.

As with monotherapy drugs, combined incubation of atorvastatin with ezetimibe and rosuvastatin with ezetimibe was able to inhibit the stimulatory effect of 25-OHC and decrease ICAM-1 mRNA and protein levels. These findings are in line with literature data. Indeed, 90-day administration of atorvastatin combined with ezetimibe (40 + 10 mg/day) was associated with a significant reduction in serum levels of ICAM-1 and hsCRP in patients with hypercholesterolemia, but no such effect was observed for ezetimibe monotherapy (10 mg/day) [32]. Similarly, a low dose of rosuvastatin combined with ezetimibe (5 mg + 10 mg/day) can exert similar anti-inflammatory effects on carotid plaque observed in the 18FDG PET/CT study of patients with acute coronary syndrome, as high dose rosuvastatin treatment (20 mg/day) [33].

The main limitation of our work is the high concentrations of the drugs used. However, the concentrations of atorvastatin (5 μM; 2793 ng/mL), rosuvastatin (10 μM; 4815 ng/mL) and ezetimibe (1.22 μM; 500 ng/mL) used in the present study correspond to those used in similar *in vitro* models [34–37]. A rosuvastatin plasma concentration of 10 μM (4815 ng/mL) has been found to correspond to the blood level in patients treated with a high therapeutic dose (40 mg) [38], and the highest plasma concentration measured after 20 mg atorvastatin single oral administration was 1 μM [39]. The highest recommended atorvastatin dose is 80 mg, which probably corresponds approximately 4 μM, and is slightly lower than the one used herein. Ezetimibe was used at a concentration of 1.22 μM (500 ng/mL). Ezetimibe has been found to directly attenuate platelet activation and demonstrate significant endothelial cell mediated effects on selected markers of atherosclerosis at a concentration of 100 ng/mL and 1000 ng/mL (0.24 and 2.44 μM) [37]. Literature data show that a healthy patient after an oral dose of 10 mg achieves a plasma concentration C_MAX_ = 70.6–73.6 ng/mL [40].

## Conclusions

Our study shows that 25-hydroxycholesterol can damage endothelial integrity and affect both tight junctions and intercellular adhesion molecules in endothelial cells. Rosuvastatin may exert a greater effect on endothelial integrity than atorvastatin, through increasing of ZO-1, OCLN and reducing of ICAM-1 mRNA and protein levels. Combined incubation of HUVEC demonstrated worse effects on the expression of intercellular junction genes and proteins than these drugs separately.

## Materials and methods

### Chemicals

Human umbilical vascular endothelial cells (HUVEC), trypsin with EDTA, trypsin neutralizing solution, endothelial cell growth medium-2 (EGM-2) with hydrocortisone, hFGF-B, vascular endothelial growth factor (VEGF), R3-IGF-1, ascorbic acid, hEGF, GA-1000, and heparin, fetal bovine serum (FBS), were from Lonza (Switzerland). Atorvastatin, rosuvastatin, ezetymibe, 25-hydroxycholesterol (25-OHC) and using primers from Sigma-Aldrich (USA). RNeasy Mini Kit was from Qiagen (Germany). High-Capacity cDNA Kit and SYBR-Green PCR Mastermix were from Applied Biosystems (USA). Other chemicals were from Roth (Germany) and POCh (Poland) and were of analytical grade.

### Cells treatment in the viability level and gene expression

Human umbilical vascular endothelial cells (HUVEC) were cultured in endothelial cell growth medium-2 (EGM-2) supplemented with 10% fetal bovine serum (FBS), hydrocortisone, hFGF-B, vascular endothelial growth factor (VEGF), R3-IGF-1, ascorbic acid, hEGF, GA-1000, heparin and penicillin (100 U/mL), and streptomycin (100 μg/mL) at 37°C, 5% CO2. After reaching 80%–90% confluence, the HUVEC were trypsinized with 0.05% trypsin with 0.02% EDTA for three minutes and then neutralized by trypsin-neutralizing solution for further experiments.

Both trypsinized HUVEC were separately seeded on 24-well plates at a density of 100,000 cells per well in 600 μl proper medium. After reaching 80–90% confluence, the HUVECs were stimulated with 25-hydroxycholesterol (24.83 μM; 10 μg/mL) for four hours. After incubation, the cells were centrifuged, the compound was discarded, and the cells were stimulated with atorvastatin (5 μM; 2793 ng/mL), rosuvastatin (10 μM; 4815 ng/mL) and ezetymibe (1.22 μM; 500 ng/mL) for 24 hours. After incubation, the cells were centrifuged, the compounds were discarded, and the cells were resuspended in EGM-2 medium.

The viability of the cells was over 97% (S1 Table), according to the Trypan Blue dye exclusion test. Data is expressed as mean ± SD.

### Institutional review board statement

The study was conducted according to the guidelines of the Declaration of Helsinki and approved by Ethics Committee of Medical University of Lodz (RNN/79/21/KE).

### Cell culture in the Real-time Cell Electric Impedance Sensing system (RTCA-DP, xCELLigence)

The RTCA-DP xCELLigence system (Roche Applied Science, Germany), which operates by tracking electrical impedance signals, enables the cell growth status to be monitored in real time on microelectrode-coated plates. The impedance readout is expressed in arbitrary units such as cell index (CI), reflecting changes in barrier properties, monolayer permeability, cell number, viability and adhesion and morphology. The normalized cell index (nCI) is calculated by dividing CI at the normalized time by the original CI value. The rate of cell growth was determined by calculating the slope of the line between two given time points. The integrated software allows data to be collected every minute for any period of time. In the presented study, the impedance measurement system was used for the dynamic and qualitative analysis of HUVEC cells.

The trypsinized HUVEC cells were separately seeded on E-16 plates at a density of 10,000 cells per well in proper media and CI changes were observed. After reaching 80–90% confluence (24 hours after seeding the cells). Data for cell adherence were normalized (nCl) before incubation with compounds. The HUVECs were stimulated with 25-hydroxycholesterol (24.83 μM; 10 μg/mL) for four hours. After incubation, the cells were centrifuged, the compound was discarded, and the cells were stimulated with atorvastatin (5 μM; 2793 ng/mL), rosuvastatin (10 μM; 4815 ng/mL) and ezetymibe (1.22 μM; 500 ng/mL) for 12 and 24 hours.

### Gene expression

RNA was extracted with the RNeasy Mini Kit (Qiagen, Germany), following the manufacturer’s instructions. RNA samples with a 260/280 nm ratio in the range of 1.8–2.0 were used for further analysis. cDNA synthesis was performed using a High-Capacity cDNA Kit (Applied Biosystems, USA). The cDNA was quantified by real-time PCR using SYBR-Green PCR Mastermix, purchased from Applied Biosystems (USA). G Gene expression was normalized to the expression of a housekeeping gene (EF1-α) and was presented as relative gene expression. The 2ΔCt (Ctgene–CtEF1-α) method was used to calculate the expression levels of studied genes. The 2-ΔCt ×100 values were re-calculated into relative copy number values (Livak and Schmittgen 2001). Primers listed in S2 Table were designed utilizing Primer-BLAST NCBI—NIH website: https://www.ncbi.nlm.nih.gov/tools/primer-blast/, and DNA sequences of selected genes were obtained using the NCBI Reference Sequences database: https://www.ncbi.nlm.nih.gov/pubmed/.

### Enzyme-linked immunosorbent assay (ELISA) for ZO-1, OCLN and ICAM-1

The presence of ZO-1, OCLN and ICAM-1 levels in the HUVEC supernatants were assessed by enzyme-linked immunosorbent assay (ELISA) using an ELISA ST-360 microplate reader (450nm and 630nm) according to the manufacturer’s protocol (Cloud-Clone, Katy, USA). The range of ZO-1 and OCLN ELISA was 0.156–10 ng/mL, ICAM-1 ELISA was 78–5000 pg/mL.

### Statistical analysis

The statistical analysis was performed with STATISTICA 13.1 data analysis software (2000 Stat-Soft, Inc., Tulsa, USA). The normality of distribution was checked using the Shapiro-Wilk test, and the homogeneity of variance by the Brown-Fisher test. Statistical analysis was conducted using the one-way analysis of variance (ANOVA) followed by Tukey’s *post hoc* multiple comparisons procedure. The difference was considered to be significant for P<0.05. The individual analysis was performed in nine-four independent experiments, while each experiment was repeated twice or three times depending on the method.

## Supporting information

S1 Fig (TIF)

S1 Table The viability of human HUVECs was determined by Trypan Blue dye exclusion test. The HUVECs were induced by 25-hydroxycholesterol (10 μg/mL), atorvastatin (5 μM; 2793 ng/mL), rosuvastatin (10 μM; 4815 ng/mL) and ezetymibe (1.22 μM; 500 ng/mL). Mean ± SD was calculated from nine individual experiments. Significant differences from negative controls were recorded at *P < 0.05. Statistical analysis was conducted using one-way ANOVA and Tukey’s test *a posteriori*.
(DOCX)

S2 Table The primers used in the real-time PCR. (DOCX)
