# Supplementary figures and images for: A comparison of the effects of monotherapy with rosuvastatin, atorvastatin or ezetimibe versus combination treatment with rosuvastatin-ezetimibe and atorvastatin-ezetimibe on the integrity of vascular endothelial cells damaged by oxidized cholesterol
Source: PLoS One. 2021 Sep 7;16(9):e0256996. doi: 10.1371/journal.pone.0256996 (PMC8423268; doi:10.1371/journal.pone.0256996)

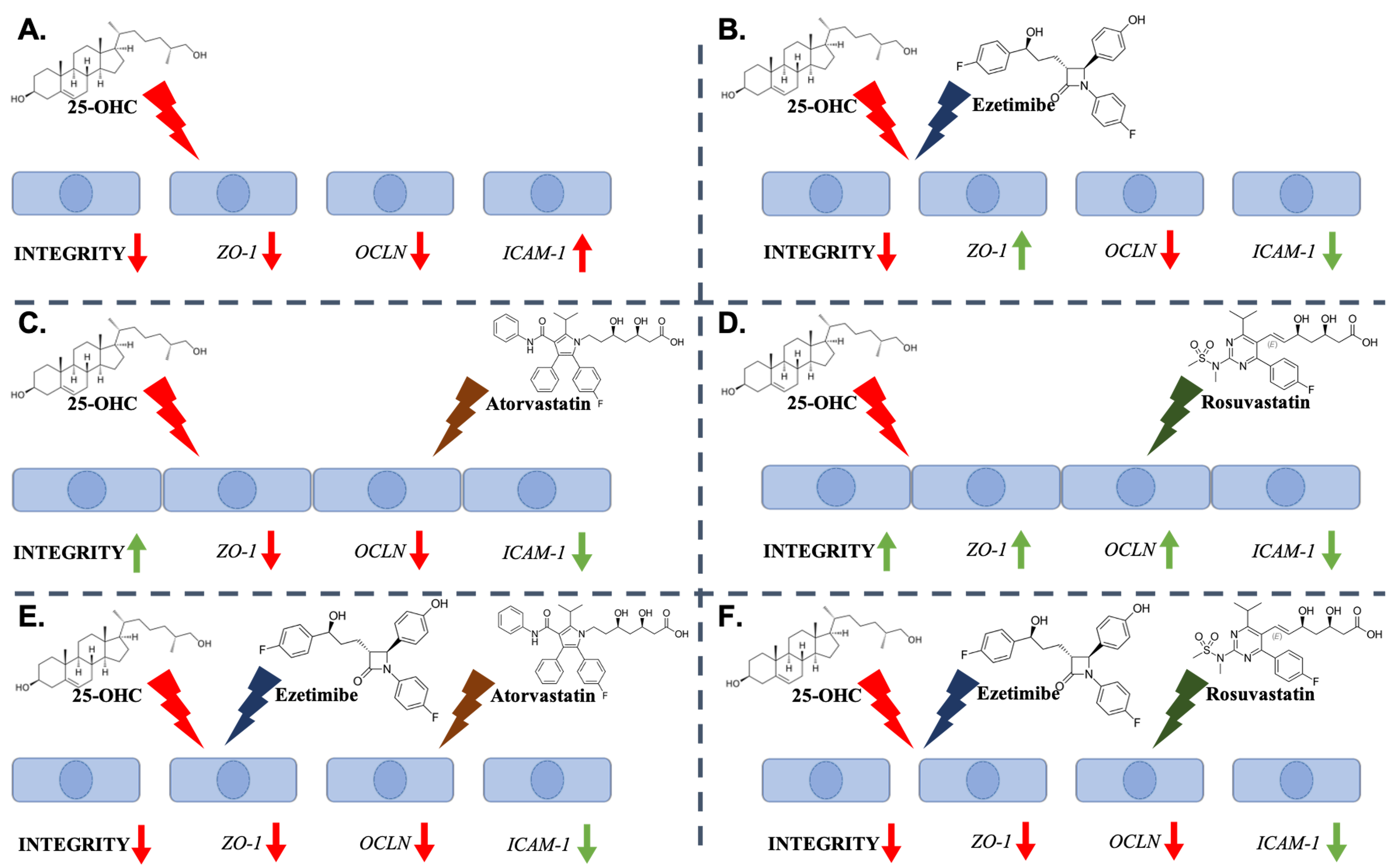

Supplement: S1 Fig — (TIF) [file pone.0256996.s001.tif]
