# Supplementary material for: A comparison of the effects of monotherapy with rosuvastatin, atorvastatin or ezetimibe versus combination treatment with rosuvastatin-ezetimibe and atorvastatin-ezetimibe on the integrity of vascular endothelial cells damaged by oxidized cholesterol
Source: PLoS One. 2021 Sep 7;16(9):e0256996. doi: 10.1371/journal.pone.0256996 (PMC8423268; doi:10.1371/journal.pone.0256996)
Supplement: S1 Table — The HUVECs were induced by 25-hydroxycholesterol (10 μg/mL), atorvastatin (5 μM; 2793 ng/mL), rosuvastatin (10 μM; 4815 ng/mL) and ezetymibe (1.22 μM; 500 ng/mL). Mean ± SD was calculated from nine individual experiments. Significant differences from negative controls were recorded at *P < 0.05. Statistical analysis was conducted using one-way ANOVA and Tukey’s test a posteriori. (DOCX) [file pone.0256996.s002.docx]

**Supplementary Table 1. The viability of human HUVECs was determined by Trypan Blue dye exclusion test.** The HUVECs were induced by 25-hydroxycholesterol (10 µg/mL), atorvastatin (5 μM; 2793 ng/mL), rosuvastatin (10 μM; 4815 ng/mL) and ezetymibe (1.22 μM; 500 ng/mL). Mean ± SD was calculated from nine individual experiments. Significant differences from negative controls were recorded at *P < 0.05. Statistical analysis was conducted using one-way ANOVA and Tukey’s test *a posteriori*.

| **Compounds** | **Concentration** | **Cell viability [%]** | **ANOVA I** |
| --- | --- | --- | --- |
| Control | 0 µg/mL | 98.1 ± 1.1 | - |
| 25-hydroxycholesterol | 24.83 μM  (10 µg/mL) | 97.8 ± 1.8 | P > 0.05 |
| Atorvastatin | 5 μM (2793 ng/mL) | 98.5 ± 1.5 | P > 0.05 |
| Rosuvastatin | 10 μM  (4815 ng/mL) | 99.1 ± 1.0 | P > 0.05 |
| Ezetymibe | 1.22 μM  (500 ng/mL) | 97.9 ± 1.4 | P > 0.05 |
| Atorvastatin + Ezetymibe | 5 μM + 1.22 μM  (2793 ng/mL + 500 ng/mL) | 97. 4 ± 1.1 | P > 0.05 |
| Rosuvastatin + Ezetymibe | 10 μM + 1.22 μM  (4815 ng/mL + 500 ng/mL) | 98.0 ± 1.2 | P > 0.05 |
