# Supplementary material for: A comparison of the effects of monotherapy with rosuvastatin, atorvastatin or ezetimibe versus combination treatment with rosuvastatin-ezetimibe and atorvastatin-ezetimibe on the integrity of vascular endothelial cells damaged by oxidized cholesterol
Source: PLoS One. 2021 Sep 7;16(9):e0256996. doi: 10.1371/journal.pone.0256996 (PMC8423268; doi:10.1371/journal.pone.0256996)
Supplement: S2 Table — (DOCX) [file pone.0256996.s003.docx]

**Supplementary Table 2.** **The primers used in the Real-Time PCR.**

| **Gene** | **Primer** | **Nucleotide sequence 5’-3’** |
| --- | --- | --- |
| *ICAM-1* | sense  anti-sense | CCCATTATGACTGCGGCTGCTA  AGGCCACCCCAGAGGACAAC |
| *OCLN* | sense  anti-sense | GAT GAG CAG CCC CCC AAT  GGT GAA GGC ACG TCC TGT GT |
| *ZO-1* | sense  anti-sense | ACAGTGCCTAAAGCTATTCCTGTGA  TCGGGAATGGCTCCTTGAG |
| **Housekeeping**  **gene** | |  |
| *EF1-α* | sense  anti-sense | CTG AAC CAT CCA GGC CAA AT  GCC GTG TGG CAA TCC AAT |
